# Supplementary material for: Background colour preference in juvenile European lobster (Homarus gammarus)
Source: Biol Open. 2026 May 11;15(5):bio062203. doi: 10.1242/bio.062203 (PMC13225228; doi:10.1242/bio.062203)
Supplement: Supplementary information [file biolopen-15-062203-s1.pdf]

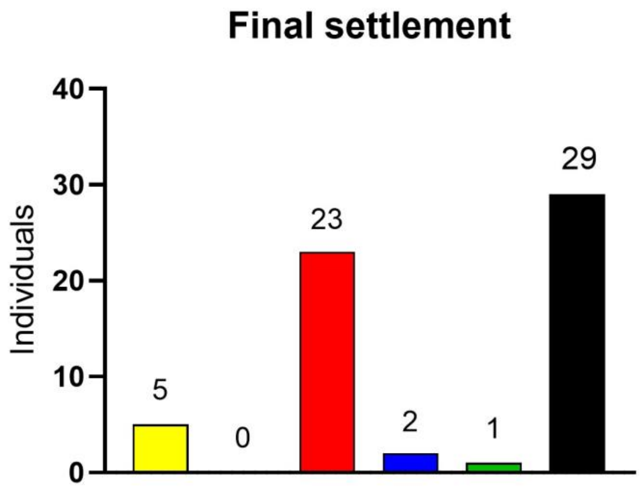

Fig. S1. Final colour settlement in sub-trial 1. N = 60. No statistical comparison was conducted as these values represent cumulative data across all individuals.

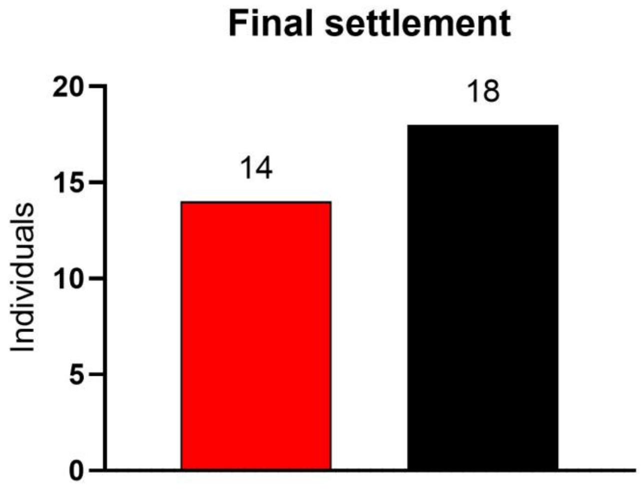

Fig. S2. Final colour settlement in sub-trial 2. N = 30. No statistical comparison was conducted as these values represent cumulative data across all individuals.

Biology Open • Supplementary information
